# Alkaline post-incubation improves the saccharification of poplar after hydrogen peroxide–acetic acid pretreatment

**DOI:** 10.1186/s13068-021-01999-7

**Published:** 2021-07-02

**Authors:** Peiyao Wen, Ying Zhang, Junjun Zhu, Yong Xu, Junhua Zhang

**Affiliations:** 1 Jiangsu Co-Innovation Center of Efficient Processing and Utilization of Forest Resources, College of Chemical Engineering, Nanjing Forestry University, Nanjing, 210037 China; 2 Key Laboratory of Forestry Genetics & Biotechnology (Nanjing Forestry University), Ministry of Education, Nanjing, 210037 China; 3 College of Forestry, Northwest A&F University, Yangling, Shaanxi, 712100 China

**Keywords:** Poplar, Hydrogen peroxide–acetic acid pretreatment, Alkaline incubation, Deacetylation, Enzymatic hydrolysis

## Abstract

**Background:**

Hydrogen peroxide–acetic acid (HPAA) is widely used in pretreatment of lignocellulose because it has a good capability in selective delignification. However, high concentration (more than 60%) of HPAA increases the cost of pretreatment and the risk of explosion. In this work, alkaline post-incubation was employed to decrease the HPAA loading and improve the saccharification of poplar.

**Results:**

Pretreatment with 100% HPAA removed 91.0% lignin and retained 89.9% glucan in poplar. After poplar was pretreated by 100% HPAA at 60 °C for 2 h, the glucan conversion in enzymatic hydrolysis by cellulase increased to 90.1%. Alkaline incubation reduced the total lignin, surface lignin, and acetyl group of HPAA-pretreated poplar. More than 92% acetyl groups of HPAA-pretreated poplar were removed by alkaline incubation with 1.0% NaOH at 50 °C for 1 h. After incubation of 60% HPAA-pretreated poplar with 1.0% NaOH, the glucan conversion enhanced to 95.0%. About 40% HPAA loading in pretreatment was reduced by alkaline incubation without the decrease of glucose yield.

**Conclusions:**

Alkaline post-incubation had strong ability on the deacetylation and delignification of HPAA-pretreated poplar, exhibiting a strong promotion on the enzymatic hydrolysis yield. This report represented alkaline incubation reduced the HPAA loading, improved pretreatment safety, exhibiting excellent potential application in saccharification of poplar.

**Supplementary Information:**

The online version contains supplementary material available at 10.1186/s13068-021-01999-7.

## Background

Poplar as a lignocellulosic material is widely used in biomass conversion [1]. Pretreatment technology is a key step for biomass conversion to produce biofuel [2–4]. The suitable pretreatment method of biomass can break the rigid structure of poplar, which is beneficial to the subsequent enzymatic hydrolysis [2–5]. However, poplar has higher lignin content and stronger physical resistance, which limit the enzymatic hydrolysis of poplar [6]. Therefore, it is necessary to remove the lignin of poplar by pretreatment to overcome the recalcitrance in enzymatic hydrolysis.

Recently, hydrogen peroxide–acetic acid (HPAA) is used in pretreatment because it has a good capability in selective delignification [7–10]. More than 98.1% lignin was removed from pine wood by 100% HPAA at 80 °C [10]. 100% HPAA pretreatment can remove 90.3% lignin of Jerusalem artichoke and improve enzymatic hydrolysis yield to 86.0% [9]. However, after the acetic acid-pretreated poplar was pretreated with 100% HPAA at 60 °C, the acetyl group increased from 5.9% to 8.0% [11]. The acetyl groups in glucan and xylan impacts its hydrolysis by cellulase or xylanase due to the steric hindrance of the acetyl groups [12–14]. Pretreatment with 0.1% sodium hydroxide can increase the glucose yield of HPAA-pretreated poplar from 67.2% to 74.4% [11]. However, the effect of sodium hydroxide incubation on the removal of acetyl in poplar has not been investigated. And the effect of sodium hydroxide concentration on surface characterization and digestibility of HPAA-pretreated poplar needs further clarification.

Normally, the high HPAA concentration (more than 60%) results in the relatively high costs of HPAA pretreatment [9–11]. Moreover, higher HPAA concentration in HPAA pretreatment faces explosion danger. In HPAA system, the formation of peracetic acid is the main reaction and the degradation of hydrogen peroxide is the side reaction [15, 16]. Pretreatment with higher HPAA concentration can form more peracetic acid, which result in the pretreatment facing explosion danger [15]. Hydrogen peroxide with high concentration with can release lots of oxygen and largely increase the pressure of pretreatment system, which put the HPAA pretreatment at the risk of explosion. Both the formation of peracetic acid and the degradation of hydrogen peroxide require the reduction of HPAA concentration. Hence, the second step pretreatment after HPAA pretreatment is proposed to increase digestibility and reduce the HPAA concentration. Alkaline incubation has been widely used in the second step pretreatment of lignocellulose [17–19]. Both the lignin and acetyl groups retained in HPAA-A pretreated lignocellulose could affect enzymatic hydrolysis. However, combined HPAA pretreatment with alkali pretreatment has not been reported and the effect of alkaline incubation on reducing HPAA loading. The effects of residual lignin and acetyl group in HPAA-A pretreated lignocellulose on enzymatic hydrolysis were not clear.

Herein, the effects of HPAA concentration on lignin removal and enzymatic hydrolysis of poplar were explored. Then, 0.1% and 1.0% sodium hydroxide were used to investigate the effects of alkaline post-incubation on the removal of acetyl group in HPAA-pretreated poplar. Effects of acetyl and lignin contents on the enzymatic hydrolysis of poplar after HPAA pretreatment and alkaline incubation were evaluated. The potential of alkaline post-incubation to decrease the cost and improve the safety of HPAA pretreatment was discussed.

## Results and discussion

### HPAA pretreatment

#### Component analysis

After poplar was pretreated by 40%, 60%, 80% and 100% HPAA, the lignin contents of poplar decreased from 27.9% to 26.5%, 19.8%, 7.7% and 4.5%, respectively (Table 1). Most glucan (89.9%–98.7%) were retained after HPAA pretreatment. More than 82.0% lignin in poplar was removed and 92.1% glucan of poplar was retained after 80% HPAA pretreatment. This data showed that HPAA pretreatment has strong glucan retention capacity and selectively delignification capability [11].

**Table 1:** Chemical compositions of poplar after pretreated with 40%–100% HPAA (v/v) at 60 °C for 2 h, expressed as percentage of dry matter

Pretreatment label	Glucan (%)	Xylan (%)	Lignin (%)	Acetyl (%)	Solid recovery (%)	Removal		
						Glucan (%)	Xylan (%)	Lignin (%)
Raw	43.4 ± 0.0	17.4 ± 0.2	27.9 ± 0.3	3.7 ± 0.2	–	–	–	–
HPAA_40_	47.4 ± 0.1	15.7 ± 0.3	26.5 ± 0.4	5.1 ± 0.3	90.2	1.3	18.7	14.3
HPAA_60_	55.0 ± 0.2	18.1 ± 0.1	19.8 ± 0.2	5.8 ± 0.3	72.4	8.3	24.4	48.6
HPAA_80_	63.7 ± 0.7	20.0 ± 0.3	7.7 ± 0.0	5.5 ± 0.7	62.7	7.9	27.7	82.8
HPAA_100_	70.8 ± 0.6	19.5 ± 0.2	4.5 ± 0.5	5.9 ± 0.7	55.1	10.1	38.1	91.2

HPAA pretreatment increased the acetyl group of poplar sample from 3.7% to 5.1%–5.9% (Table 1). The increase of acetyl content was consistent with the results in other report [11]. The formation of acetyl group was attributed to the reason that hydroxyl group of cellulose and xylan was esterified by acetic acid under the catalysis of sulfuric acid [12–14].

HPAA pretreatment increased the acetyl content of poplar (Table 1). The grafting of extensive acetyl groups on the cellulose or hemicellulose in poplar hindered the adsorption of cellulases, thereby decreasing the monosaccharides yield of saccharification process [13, 14, 19]. Therefore, it was necessary further remove the acetyl group of HPAA-pretreated poplar.

#### XPS analysis

The higher O/C ratio reflects lower lignin and extractives contents on the surface of biomass [20]. The O/C ration of HPAA_40_-pretreated poplar was 0.38, which was close to the 0.39 of non-pretreated poplar (Table 2). When the HPAA concentration was higher than 40%, the O/C ratio of poplar increased to 0.40–0.45. This data showed that pretreatment with 60%–100% HPAA could decrease the lignin or extractives (i.e., fatty acids, hydrocarbons) contents on the surface of poplar [21]. Meanwhile, the C1 peak is primarily composed of lignin and extractives [22]. With HPAA concentration increased from 40 to 100%, the C1 peak value of the poplar was decreased from 55.1% to 46.7%, which showed that increasing HPAA loading could remove the lignin from the surface of poplar [20, 22].

**Table 2:** XPS analysis of poplar after pretreated with 40%–100% HPAA (v/v) at 60 °C for 2 h

Pretreatment label	O/C	C1 (%)	C2 (%)	C3 (%)
Raw	0.39 ± 0.0	56.3 ± 0.1	31.3 ± 0.5	12.4 ± 0.4
HPAA_40_	0.38 ± 0.0	55.1 ± 0.2	33.4 ± 0.1	11.5 ± 0.3
HPAA_60_	0.40 ± 0.0	52.3 ± 0.7	34.7 ± 0.5	13.0 ± 0.1
HPAA_80_	0.45 ± 0.0	46.7 ± 0.0	37.6 ± 0.7	15.7 ± 0.7
HPAA_100_	0.43 ± 0.0	47.3 ± 0.6	37.0 ± 1.1	15.7 ± 0.5

C1 corresponds to class of carbon that corresponds to carbon atoms bonded to carbon or hydrogen (C–C or C-H)

C2 corresponds to class of carbon atoms bonded to single non-carbonyl oxygen(C–O)

C3 corresponds to class of carbon atoms bonded to a carbonyl or two non-carbonyls (C=O or O–C–O)

#### Enzymatic hydrolysis

**Fig.1:**
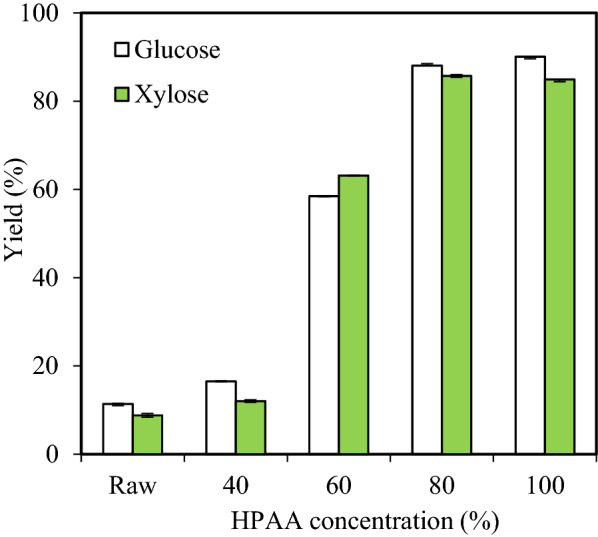
The monosaccharide yields of 40%–100% HPAA-pretreated poplar by CTec2 (10 FPU/g DM) at 50 °C and pH 5.0 for 72 h

After poplar pretreated with 40%–100% HPAA for 2 h, glucose yields of poplar by a CTec2 loading of 10 FPU/g DM increased from 11.4% to 16.5%–90.1% (Fig. 1). This result might be attributed to the lignin removal of poplar by HPAA pretreatment [9, 11]. With HPAA concentration increased from 40% to 100%, the glucose and xylose yields of poplar samples improved from 16.5% and 12.0% to 90.1% and 84.9%, respectively (Fig. 1). The glucose yield of HPAA_80_-pretreated poplar was 88.1%, which was slightly lower than those yields of HPAA_100_-pretreated poplar. This data showed that excessive HPAA loading (more than 80%) cannot greatly improve the hydrolysis yield of poplar.

### Alkaline post-incubation

#### Component analysis

After alkaline post-incubation with 0.1% sodium hydroxide, 15.6%–37.8% acetyl groups of HPAA-pretreated poplar were removed (Table 3). Meanwhile, 22.1%–64.5% lignin of HPAA-pretreated poplar was removed. The acetyl and lignin removals of HPAA_100_-pretreated poplar were higher than those of HPAA_60_- and HPAA_80_-pretreated poplar. Those data suggested that the acetyl and lignin of poplar pretreated with higher HPAA concentration might be easier removed by 0.1% sodium hydroxide.

**Table 3:** Chemical compositions of poplar after HPAA–sodium hydroxide (HPAA–SH) process, expressed as percentage of dry matter

Pretreatment label	Glucan (%)	Xylan (%)	Lignin (%)	Acetyl (%)	Solid recovery (%)	Removal			
						Glucan (%)	Xylan (%)	Lignin (%)	Acetyl (%)
HPAA_40_–SH_0.1_	52.0 ± 0.9	17.8 ± 0.4	19.7 ± 0.1	4.8 ± 0.2	87.7	4.0	0.2	34.8	16.8
HPAA_60_–SH_0.1_	56.3 ± 0.2	18.9 ± 0.3	16.8 ± 0.1	5.3 ± 0.2	92.0	5.8	4.1	22.1	15.6
HPAA_80_–SH_0.1_	68.0 ± 0.5	20.0 ± 0.3	4.1 ± 0.0	5.2 ± 0.1	89.5	4.5	10.5	52.5	15.6
HPAA_100_–SH_0.1_	71.9 ± 0.6	18.4 ± 0.4	1.9 ± 0.0	4.3 ± 0.3	85.3	13.4	19.4	64.5	37.8
HPAA_40_–SH_1.0_	56.1 ± 0.1	18.4 ± 0.0	18.8 ± 0.2	0.5 ± 0.0	79.8	5.7	6.4	43.6	92.1
HPAA_60_–SH_1.0_	68.2 ± 0.4	20.1 ± 0.2	6.1 ± 0.0	0.5 ± 0.0	76.2	5.5	15.4	76.7	93.9
HPAA_80_–SH_1.0_	76.7 ± 2.5	20.0 ± 0.2	1.4 ± 0.0	0.4 ± 0.0	77.5	6.7	22.4	85.4	94.7
HPAA_100_–SH_1.0_	80.5 ± 2.6	17.7 ± 0.8	0.2 ± 0.0	0.4 ± 0.0	75.7	14.0	31.5	96.5	95.0

In HPAA–SH process, HPAA pretreatment conditions were 40%–100% HPAA (v/v) at 60 °C for 2 h and alkaline post-incubation conditions were 0.1% and 1.0% sodium hydroxide at 50 °C for 1 h. The removal of HPAA–SH-pretreated poplar was based on the HPAA-pretreated poplar

However, when the sodium hydroxide concentration increased to 1.0%, the acetyl groups of HPAA–SH_1.0_-pretreated poplar were greatly decreased to 0.4%–0.5% (Table 3). This data showed that 1.0% sodium hydroxide was very effective at the deacetylation of the HPAA-pretreated poplar. Meanwhile, 43.6%–96.5% lignin of HPAA-pretreated poplar was removed by 1.0% sodium hydroxide. These data suggested that 1.0% sodium hydroxide pretreatment had relatively strong delignification and deacetylation ability on HPAA-pretreated poplar.

#### XPS analysis

After alkaline post-incubation with 0.1% sodium hydroxide, the O/C ratio of HPAA-pretreated poplar improved to 0.39–0.47 (Table 4). A high O/C suggests higher carbohydrate content is cover on the surface of biomass, while a low O/C shows more lignin [23]. This data indicated that alkaline post-incubation reduced the surface lignin of HPAA-pretreated poplar [21, 24]. This result was consistent with the previous report that sodium hydroxide treatment decreases the surface lignin of hybrid *Pennisetum* [24]. When the sodium hydroxide concentration was increased to 1.0%, the O/C ratios of poplar of HPAA-pretreated poplar were increased to 0.40–0.50, which were higher than those of HPAA–SH_0.1_-pretreated poplar. It implied that post-incubation with 1.0% sodium hydroxide could remove more surface lignin of HPAA-pretreated poplar than that with 0.1% sodium hydroxide. The decrease of surface lignin would be beneficial to the following hydrolysis of HPAA-pretreated poplar as surface lignin limits the accessibility of cellulase to cellulose in poplar [25].

**Table 4:** XPS analysis of HPAA–SH-pretreated poplar

Pretreatment label	O/C	C1 (%)	C2 (%)	C3 (%)
HPAA_40_–SH_0.1_	0.39 ± 0.0	54.4 ± 0.3	32.9 ± 0.6	12.7 ± 0.3
HPAA_60_–SH_0.1_	0.42 ± 0.0	49.3 ± 0.1	36.0 ± 0.5	14.6 ± 0.4
HPAA_80_–SH_0.1_	0.47 ± 0.0	43.8 ± 0.3	41.5 ± 0.0	14.7 ± 0.3
HPAA_100_–SH_0.1_	0.46 ± 0.0	45.6 ± 0.0	38.1 ± 0.3	16.3 ± 0.3
HPAA_40_–SH_1.0_	0.40 ± 0.0	53.4 ± 0.2	34.9 ± 0.8	11.7 ± 0.6
HPAA_60_–SH_1.0_	0.44 ± 0.0	49.4 ± 0.1	37.5 ± 0.2	13.1 ± 0.1
HPAA_80_–SH_1.0_	0.50 ± 0.0	43.6 ± 0.1	41.5 ± 0.1	15.0 ± 0.0
HPAA_100_–SH_1.0_	0.50 ± 0.0	40.6 ± 0.2	43.0 ± 0.3	16.5 ± 0.0

In HPAA–SH process, HPAA pretreatment conditions were 40%–100% HPAA (v/v) at 60 °C for 2 h and alkaline post-incubation conditions were 0.1% and 1.0% sodium hydroxide at 50 °C for 1 h

#### Enzymatic hydrolysis

**Fig. 2:**
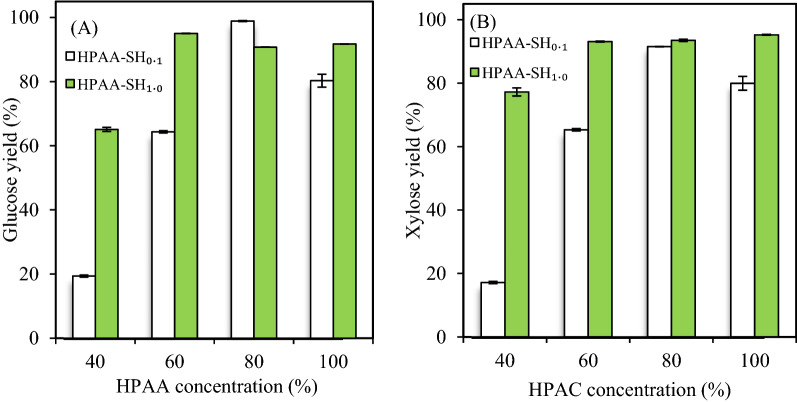
Effects of alkaline post-incubation on the glucose **(A)** and xylose **(B)** yields of 40%–100% HPAA-pretreated poplar by CTec2 (10 FPU/g DM) at 50 °C and pH 5.0 for 72 h

After alkaline post-incubation with 0.1% sodium hydroxide, the glucose yields of HPAA_40_-, HPAA_60_-, and HPAA_80_-pretreated poplar samples increased from 16.5%–88.1% to 19.3%–98.9% (Fig. 2A). This increase was due to the delignification and deacetylation by alkaline incubation [12, 26]. Unexpectedly, the incubation with 0.1% NaOH decreased the glucose yield of HPAA_100_-pretreated poplar from 90.1% to 80.3%. Meanwhile, the glucose yield (90.8%) of HPAA_80_–SH_1.0_-pretreated poplar was lower than that of HPAA_80_–SH_0.1_-pretreated poplar (98.9%). This phenomenon has been confirmed by many other authors, which exhibited further delignification of sample by alkaline incubation cannot increase efficiency of enzymatic hydrolysis anymore [27, 28]. These results might be due to that higher sodium hydroxide and HPAA concentration removed much amorphous cellulose [11] in poplar because the amorphous cellulose was easier to hydrolysis [29]. Furthermore, excessive removal of lignin during pretreatment results in the aggregation of cellulose, which could negatively affect the surface accessibility [29, 30]. Hence, the aggregation of cellulose by pretreatment could be a reason that higher HPAA or alkali concentrations reduced the hydrolysis yield of poplar.

**Fig.3:**
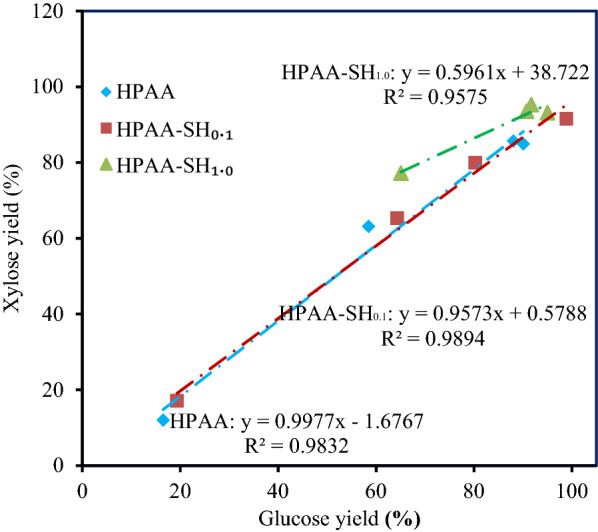
Relationship between xylose and glucose yields in the hydrolysis of 2% HPAA- and HPAA–SH-pretreated poplar

When the sodium hydroxide concentration increased to 1.0%, the glucose yields of HPAA_40_–SH_1.0_-, HPAA_60_–SH_1.0_-, HPAA_80_–SH_1.0_-, and HPAA_100_–SH_1.0_-pretreated poplar were 65.1%, 95.0%, 90.8%, and 91.7%, respectively. The glucose yields of HPAA_60_–SH_1.0_- and HPAA_100_-pretreated poplar were close (Figs. 1 and 3), which showed that alkaline incubation can reduce 40% HPAA loading in pretreatment without obvious decrease of hydrolysis yield.

Additionally, alkaline post-incubation with 1.0% sodium hydroxide greatly improved the xylose yield of HPAA-pretreated poplar from 12.0% to 85.7% to 77.0%–95.2% (Fig. 2B). These data could be due to that alkaline post-incubation removed the acetyl group of hemicellulose in HPAA-pretreated poplar, which reduced the steric hindrance of acetyl group to xylanase in CTec2 and improved the hydrolysis efficiency of xylanase [13].

It was found that linear relationship (*R*^2^ > 0.95) existed between the glucose and xylose yields in enzymatic hydrolysis of HPAA- and HPAA–SH-pretreated poplar (Fig. 3). This result could be due to the synergistic effect of the cellulase and xylanase in CTec2 affected the enzymatic hydrolysis [31]. Higher xylose yields were got from HPAA–SH_1.0_-pretreated poplar than HPAA- and HPAA–SH_0.1_-pretreated poplar when the same amount of glucose was released. This data showed that the xylan in HPAA–SH_1.0_-pretreated poplar was more easily hydrolyzed than that xylan in HPAA- and HPAA–SH_0.1_-pretreated poplar.

## Relationship between lignin content and hydrolysis yield

In alkaline incubation process, lignin content and acetyl group are two main factors affecting enzymatic hydrolysis of pretreated lignocellulose [28, 32]. In a certain range of lignin content, the materials with lower lignin content are more easily degraded by cellulase [28]. In this work, the relationships between lignin contents and the glucose yields of HPAA- and HPAA–SH-pretreated poplar were analyzed (Additional file 1: Fig. S1). The lignin contents and the glucose yields of HPAA–SH-pretreated poplar showed lower linear correlation (*R*^2^ = 0.72–0.82) than those of HPAA-pretreated poplar (0.9). This result might be due to alkaline incubation removed excessive lignin of poplar and excessive delignification of poplar, which could not increase efficiency of enzymatic hydrolysis anymore [28].

A linear relationship (*R*^2^ > 0.94) existed between lignin content and the xylose yields in the hydrolysis of HPAA–SH_1.0_-pretreated poplar. However, the xylose yields of HPAA- and HPAA–SH_0.1_-pretreated poplar showed lower linear correlation with lignin contents. These data could be due to the presence of high acetyl contents in the HPAA- and HPAA–SH_0.1_-pretreated poplar (Tables 1 and 3), which impacted the hydrolysis of poplar xylan [12].

## Relationship between acetyl content and hydrolysis yield

After alkaline incubation with different concentrations of sodium hydroxide, the HPAA–SH-pretreated poplar samples contained different acetyl contents (Table 3). When the HPAA concentrations in pretreatment were 40% or 60%, a linear relationship (R^2^ > 0.99) between acetyl content and monosaccharides (glucose and xylose) yields existed (Additional file 1: Fig. S2). This data indicated that acetyl content played a very important role in the hydrolysis of 40% and 60% HPAA-pretreated poplar [32]. However, it was not found the linear relation between acetyl content and monosaccharide yields in 80% and 100% HPAA-pretreated poplar, which indicated that besides acetyl group, there some other factors (such as cellulase and xylanase activities in hydrolysate, inhibitor in hydrolysate, amorphous cellulose content in samples) might affect the enzymatic hydrolysis of 80% or 100% HPAA-pretreated poplar [32, 33].

## Mass balance

**Fig.4:**
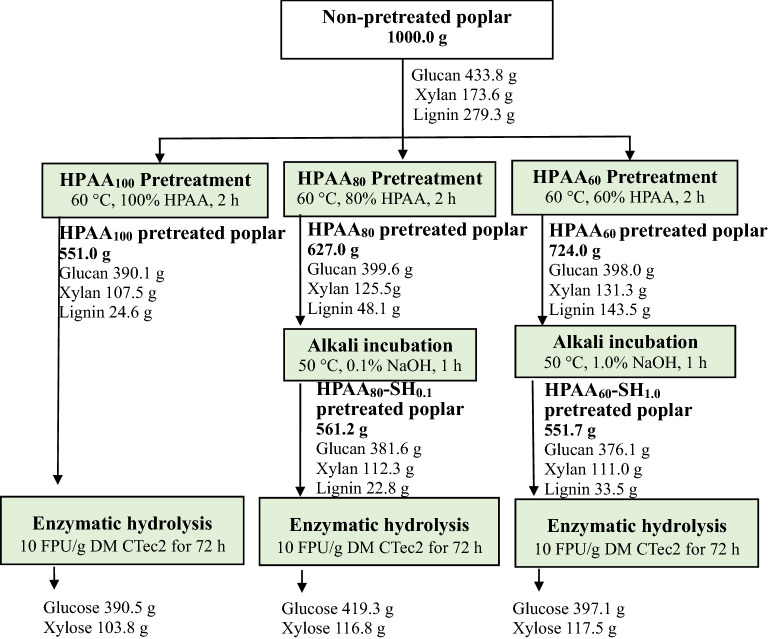
Mass balances for production of monosaccharides from poplar after HPAA_80_–SH_0.1_ and HPAA_60_–SH_1.0_ pretreatments

Figure 4 presents the mass balance of poplar after HPAA pretreatment and subsequent enzymatic hydrolysis using CTec2. After pretreatment with 100% HPAA, 390.5 g glucose and 103.8 g xylose were got from 1 000 g raw poplar by enzymatic hydrolysis. Interestingly, HPAA_60_–SH_1.0_ and HPAA_80_–SH_0.1_ pretreatments processes gave 514.6 g and 536.1 g monosaccharides (glucose and xylose) from 1 000 g raw poplar, which were higher than those obtained from HPAA_100_ pretreatment (494.3 g) without alkaline post-incubation. These results showed that alkaline incubation not only reduced the HPAA loading in the pretreatment, but also increased monosaccharide yields from poplar. In previous report, after poplar pretreated with 80% HPAA, only 190.0 g glucose and 86.0 g xylose were obtained from 1 000 g raw poplar by enzymatic hydrolysis [34]. Compared with the previous report [34], the higher monosaccharide yields obtained in this work improved the economic benefit, and the relatively lower HPAA concentration (60%) improved the safety of pretreatment.

Normally, the pretreatment temperature of poplar is higher than 150 °C [35–39]. Herein, the temperatures of HPAA pretreatment and alkaline post-incubation were 60 °C and 50 °C, respectively, indicating that they were relatively mild pretreatments. Moreover, some reports obtained relatively low glucose yields (less than 65.0%) from pretreated poplar and the cellulase loading were higher than 15 FPU/g DM [40–42]. Furthermore, the enzymatic hydrolysis of HPAA-pretreated lignocelluloses needs extra cellulase or surfactant to improve hydrolysis yield [11, 43, 44]. Herein, more than 95.0% glucose yields were got from poplar and only 10 FPU CTec2 per g DM was used in the enzymatic hydrolysis without extra cellulase or surfactant. Therefore, the HPAA–SH process had a great potential to decrease the cost of monosaccharides production from poplar. To reduce the cost of the HPAA–SH pretreatment, pressure shift distillation can be employed to separate acetic acid and the peracetic acid formed in HPAA solution [45, 46]. Furthermore, the HPAA solution can be reused in the next pretreatment to reduce the cost. Sodium hydroxide solution in alkaline post-incubation can be reused in next process. Furthermore, sodium hydroxide solution also can be diluted and neutralized as a buffer used in enzymatic hydrolysis, which can eliminate washing process of poplar [18]. The process of HPAA and alkaline post-incubation reduced HPAA loading and provided a preferable feature for the production of sugars from poplar with relatively mild conditions.

## Conclusions

HPAA pretreatment removed 14.3%–91.2% lignin of poplar and increased the acetyl content to 5.1%–5.9%. More than 93.0% acetyl and 76.0% lignin contents of HPAA_60_-pretreated poplar was removed by 1.0% sodium hydroxide. Alkaline incubation reduced 40% HPAA loading in pretreatment and increased the glucose yield of HPAA_60_-pretreated poplar to 95.0%. The results in this work showed that alkaline post-incubation had strong ability on the deacetylation and delignification of HPAA-pretreated poplar, exhibiting a strong promotion on enzymatic hydrolysis yield. This report provided a scientific guidance for production of monosaccharides from poplar by HPAA pretreatment with less HPAA loading and low temperature.

## Materials and methods

### Materials

Poplar was obtained from the Nanjing Forestry University (Jiangsu, China). Cellic CTec2 (Novozymes A/S, Bagsværd, Denmark) had an activity of 123.0 filter paper units (FPU)/mL (176.2 mg protein/mL). Acetic acid and sodium hydroxide used in this work were purchased from Guanghua sci-tech Co., Ltd (Guangdong, China).

### HPAA pretreatment

The HPAA solution was prepared by mixing hydrogen peroxide (30%, w/w) and acetic acid (99%, w/w) at a ratio of 1:1 (v/v) [11]. HPAA pretreatments were investigated at 60 °C with 40%–100% (v/v). The pretreatment with 40%, 60%, 80%, and 100% HPAA were labeled as HPAA_40_, HPAA_60_, HPAA_80_, and HPAA_100_, respectively. All pretreatments were performed at a solid-to-liquid ratio of 1:10 (w/v) for 2 h with 100 mM H_2_SO_4_ as a catalyst. The solid residues were separated by filtration and washed extensively with distilled water until the wash water had a neutral pH, then stored at − 20 °C for chemical composition analysis and enzymatic hydrolysis. All pretreatments were carried out in duplicate and the average values are presented.

### Alkaline post-incubation

HPAA-pretreated samples were employed for the subsequent alkaline post-incubation. The alkaline post-incubation was performed at 50 °C for 1 h with a solid-to-liquid ratio of 1:10 (w/v) by 0.1% and 1.0% sodium hydroxide, respectively, which were labeled as HPAA–SH_0.1_ and HPAA–SH_1.0_. All experiments were carried out in duplicate and the average values are presented.

### Enzymatic hydrolysis

Enzymatic hydrolysis of poplar samples was conducted in 10-mL test tubes (601,051–1, Biosharp, Hefei, China) with a working volume of 3.0 mL in 50 mM sodium citrate buffer (pH 5.0) at 50 °C and 200 rpm. Poplar samples were loaded at 2% (w/v) with a Cellic CTec2 loading of 10 FPU/g DM for 72 h. All hydrolysis experiments were carried out in duplicate and the average values are presented.

### Analytical methods

Monosaccharides were analyzed by the method as described previously [21]. The contents of glucan, xylan and lignin of poplar were determined by the National Renewable Energy Laboratory analytical procedure [47]. The acetyl contents of poplar solids were determined as per NREL Laboratory Analytical Procedure 002 method using glacial acetic acid as a calibration standard [11]. XPS analysis was consistent with the method previously reported [48]. All experiments were carried out in duplicate and the average values are presented.

## Calculations

### Calculations

The recovery and removal of each component were calculated by the following formula:

1 $${\text{Soild}\;\text{recovery}}\left( \% \right) = \frac{{{W_{\text{after}\; \text{pretreatment}}}}}{{{W_{\text{before}\; \text{pretreatment}}}}} \times 100$$

where W_before pretreatment_ and W_after pretreatment_ were the weight of poplar before and after pretreatment.

2 $${\text{Removal}}\;\left( \% \right) = \left( {1 - \frac{{{{\text{W}}_{{\text{component}}\;{\text{after}}\;{\text{pretreatment}}}}\;}}{{{{\text{W}}_{{\text{component}}\;{\text{before}}\;{\text{pretreatment}}}}}}} \right) \times 100$$

where W_component before pretreatment_ and W_component after pretreatment_ were the weight of the components (cellulose, xylan, and lignin) in poplar before and after pretreatment.

The glucose and xylose yields of enzymatic hydrolysis were calculated based on the following equations:

3 $${\text{Glucose}\;{\text{yield}}}\; (\% ) = \frac{{{\text{Glucose in enzymatic hydrolysate}} \times 0.9}}{{{\text{Glucan in treated poplar}}}} \times 100$$

4 $${\text{Xylose}}\,{\text{yield}}\,(\% )\, = \,\frac{{{\text{Xylose}}\,{\text{in}}\,{\text{enzymatic}}\,{\text{hydrolysate}}\, \times \,0.88}}{{{\text{Xylan}}\,{\text{in}}\,{\text{treated}}\,{\text{poplar}}}} \times \,100$$

## Supplementary Information

**Additional file 1: Fig. S1** Relationship between lignin contents and the monosaccharide yields of HPAA- and HPAA–SH-pretreated poplar. **Fig. S2** Relationship between acetyl contents and the monosaccharide yields of HPAA- and HPAA–SH-pretreated poplar.

## Data Availability

All relevant data have been included in this published article and its Additional file 1.

## Abbreviations

HPAA: Hydrogen peroxide–acetic acid
SH: Sodium hydroxide
DM: Dry mass
XPS: X-ray photoelectron spectroscopy
FPU: Filter paper unit
