# Supplementary material for: Alkaline post-incubation improves the saccharification of poplar after hydrogen peroxide–acetic acid pretreatment
Source: Biotechnol Biofuels. 2021 Jul 2;14:151. doi: 10.1186/s13068-021-01999-7 (PMC8254297; doi:10.1186/s13068-021-01999-7)
Supplement: Supplementary file 1 — Additional file 1: Fig. S1 Relationship between lignin contents and the monosaccharide yields of HPAA- and HPAA–SH-pretreated poplar. Fig. S2 Relationship between acetyl contents and the monosaccharide yields of HPAA- and HPAA–SH-pretreated poplar. [file 13068_2021_1999_MOESM1_ESM.docx]

**Alkaline post-incubation improves the saccharification of poplar after hydrogen peroxide-acetic acid pretreatment**

Peiyao Wen^a,b,c^, Ying Zhang^c^, Junjun Zhu^a,b^, Yong Xu^a,b^, Junhua Zhang^a,b,c^*

^a^ Jiangsu Co-Innovation Center of Efficient Processing and Utilization of Forest Resources, College of Chemical Engineering, Nanjing Forestry University, Nanjing 210037, China

^b^Key Laboratory of Forestry Genetics & Biotechnology (Nanjing Forestry University), Ministry of Education, Nanjing 210037, China

^c^ College of Forestry, Northwest A&F University, Yangling 712100, Shaanxi, China

Corresponding author. Tel.: +86-13770609339

E-mail address: junhuazhang@njfu.edu.cn (J. Zhang)

Table S1. The glucose and xylose yields of poplar in HPAC pretreatment liquid. The pretreatment conditions were 40%–100% HPAA (v/v) at 60 °C for 2 h.

| Pretreatment label | Glucose yield (%) | Xylose yield (%) |
| --- | --- | --- |
| HPAA_40_ | bdl | bdl |
| HPAA_60_ | 0.4 ±0.0 | 4.8 ±0.2 |
| HPAA_80_ | 0.6 ±0.0 | 4.8 ±0.2 |
| HPAA_100_ | 1.3 ±0.1 | 4.7 ±0.2 |

The bdl means the data was below detection limit. The glucose and xylose yields in HPAC pretreatment liquid were calculated based on the following equations:

$$Glucose yield \left( \% \right)=\frac{Glucose in HPAC pretreatment liquid\times0.9}{Glucan in raw poplar}\times100 (1)$$

$$Xylose yield \left( \% \right)=\frac{Xylose in HPAC pretreatment liquid\times0.88}{Xylan in raw poplar}\times100 (2)$$

Table S2. The glucose and xylose yields of poplar in alkaline incubation liquid.

In HPAA-SH process, HPAA pretreatment conditions were 40%–100% HPAA (v/v) at 60 °C for 2 h and alkaline post-incubation conditions were 0.1% and 1.0% sodium hydroxide at 50 °C for 1 h.

| Pretreatment label | Glucose yield (%) | Xylose yield (%) |
| --- | --- | --- |
| HPAA_40_-SH_0.1_ | 1.1 ±0.1 | bdl |
| HPAA_60_-SH_0.1_ | 1.6 ±0.1 | 1.0 ±0.0 |
| HPAA_80_-SH_0.1_ | 1.7 ±0.1 | 4.8 ±0.2 |
| HPAA_100_-SH_0.1_ | 3.3 ±0.2 | 5.4 ±0.3 |
| HPAA_40_-SH_1.0_ | 1.2 ±0.1 | 3.1 ±0.1 |
| HPAA_60_-SH_1.0_ | 1.7 ±0.1 | 4.8 ±0.2 |
| HPAA_80_-SH_1.0_ | 2.6 ±0.1 | 8.0 ±0.4 |
| HPAA_100_-SH_1.0_ | 3.1 ±0.1 | 11.3 ±0.5 |

The bdl means the data was below detection limit. The glucose and xylose yields in alkaline incubation liquid were calculated based on the following equations:

$$Glucose yield \left( \% \right)=\frac{Glucose in \mathrm{alkaline}incubation liquid\times0.9}{Glucan in HPAA treated poplar}\times100 (3)$$

$$Xylose yield \left( \% \right)=\frac{Xylose in \mathrm{alkaline}incubation liquid\times0.88}{Xylan in HPAA treated poplar}\times100 (4)$$

**Fig. S1** Relationship between lignin contents and the monosaccharides yields of HPAA- and HPAA-SH-pretreated poplar.

**Fig. S2** Relationship between acetyl contents and the monosaccharides yields of HPAA- and HPAA-SH pretreated poplar. The HPAA-pretreated poplar with different acetyl contents were obtained by alkaline post-incubation.
